# Resilience of local food systems and links to food security – A review of some important concepts in the context of COVID-19 and other shocks

**DOI:** 10.1007/s12571-020-01076-1

**Published:** 2020-07-11

**Authors:** Christophe Béné

**Affiliations:** International Center for Tropical Agriculture (CIAT), Cali, Colombia

**Keywords:** Food systems, Resilience, Food security, COVID-19, Shocks

## Abstract

The objective of this review is to explore and discuss the concept of local food system resilience in light of the disruptions brought to those systems by the 2020 COVID-19 pandemic. The discussion, which focuses on low and middle income countries, considers also the other shocks and stressors that generally affect local food systems and their actors in those countries (weather-related, economic, political or social disturbances). The review of existing (mainly grey or media-based) accounts on COVID-19 suggests that, with the exception of those who lost members of their family to the virus, as per June 2020 the main impact of the pandemic derives mainly from the lockdown and mobility restrictions imposed by national/local governments, and the consequence that the subsequent loss of income and purchasing power has on people’s food security, in particular the poor. The paper then uses the most prominent advances made recently in the literature on household resilience in the context of food security and humanitarian crises to identify a series of lessons that can be used to improve our understanding of food system resilience and its link to food security in the context of the COVID-19 crisis and other shocks. Those lessons include principles about the measurement of food system resilience and suggestions about the types of interventions that could potentially strengthen the abilities of actors (including policy makers) to respond more appropriately to adverse events affecting food systems in the future.

## Introduction

The impact of COVID-19 on the lives of the billions of people who are affected by the pandemic is not limited to the direct threat that the virus imposes on their health. It extents to their food security through the disruptions that it is having on local and national food systems and economies. To a large extent, COVID-19 did not reveal only the limits of our (national and international) health systems; it also illustrated the fragility of our food systems, and how easily those can be disrupted. In sum, it sheds light on the central question of the resilience of food systems and its link to people’s food and nutrition security.

The premise of this paper is the recognition that the largest part of the food and nutrition insecurity observed at the local levels (households, communities, districts levels) in low and middle income countries (LMICs) is the result of two combined and reinforcing issues: (a) *Structural issues -* In these LMICs, small-scale producers and food suppliers typically operate under extremely difficult conditions, including inadequate infrastructures (roads, power, irrigation and wholesale markets) leading to geographic and economic isolation, little opportunity to develop business, lack of access to services (training, credit, supplies) and high dependence on weather conditions (McCullough et al. 2010); (b) *Shocks and stressors -* In addition to those structural deficiencies, another large part of the current issues reflects directly the inability of the local / provincial food systems to respond and recover rapidly from the effects of shocks and stressors. When local or meso-scale shocks (drought, flood) or stressor (corruption, local insecurity, seasonal road inaccessibility) occur, those events severely affect the different actors involved in local and regional food supply chains (food producers, retailers, transporters, etc.) and prevent most of them from operating efficiently (Sabates-Wheeler et al. 2008; Harvey et al. 2014). This generally results in physical and economic disruptions of the food supply operations -leading to food shortage, food losses, or price volatility in both rural and urban areas, with short term and long-term implications for both chronic and acute hunger and malnutrition.

The disruptions of national economies following the various forms of restrictions imposed by local and/or national authorities in response to COVID-19 are an example of those shocks/stressors that affect the ability of local food systems to operate. The objective of this review is to explore and discuss the concept of “local food system resilience” in the light of the disruptions brought to those systems by the COVID-19 pandemic. We are interested here in the food systems operated in LMICs (now representing more than 6.5 billion people), and our analysis focuses on the local level, where the interactions between the different actors of the systems (producers, retailers, consumers) take place.^1^

A small body of literature is already available on the concept of food system resilience (see e.g. Pingali et al. 2005; Rotz and Fraser 2015; Tendall et al. 2015). To complement this literature we propose to build on the most recent research that was produced in the last 5 to 7 years on household resilience in the context of food security crises (e.g. Constas et al. 2014; Brück et al. 2018; Ansah et al. 2019; Béné et al. 2020) and identify what and how the lessons and principles that emerged from this new body of work can be useful in improving our understanding of food system resilience and its link to food security in the context of the COVID-19 disruptions. Our contribution will be mainly conceptual but builds on the empirical experience that we gained in the field while implementing and/or assessing resilience and food security programmes and interventions in both Africa and Asia.

## Clarifying concepts

In this paper, the term food security is used in a conventional manner, one that encompasses the four traditional dimensions of food security: food availability, food accessibility, food utilization, and stability (FAO 2008). As such, this generic definition puts emphasis on some critical aspects of the concept of food security which will be relevant for the discussion on resilience later in this paper, in particular the idea that food security cannot be achieved without some element of stability in the access to, availability of, and quality of, food, and that this stability can to some degree be linked to resilience.

Many definitions of resilience exist in the literature across the different domains where resilience is being used (see e.g. Windle 2011; Patel et al. 2017; Béné and Doyen 2018; Barasa et al. 2018). In the sphere of humanitarian and food security interventions, several of those definitions and associated frameworks are now widely referred to in both academic and practitioner communities (see, e.g., DFID 2011, USAID 2012; FAO 2013, WFP 2020). Although slightly different in their wording, they all fundamentally carry the same message: in the context of humanitarian and food security programmes, resilience is about the capacities of households and communities, to deal with adverse events in a way that does not affect negatively their long-term wellbeing and/or functioning. Constas and his colleagues from the Resilience Measurement Technical Working Group for instance defines resilience as “the capacity that ensures stressors and shocks do not have long-lasting adverse development consequences” (Constas et al. 2013, p.6).

Food systems encompass “all the elements (environment, people, inputs, processes, infrastructures, institutions, etc.) and activities that relate to the production, processing, distribution, preparation consumption [and waste management] of food, and the output of these activities, including socio-economic and environmental outcomes” (HLPE 2017, p.23, our addition). Beyond this all-embracing definition, local food systems in LMICs display other features of importance for our discussion. LMIC local food systems are both made up and benefit many of the world’s poorest citizens (Smith 1998; Gómez et al. 2013). At the production end, they includes the vast majority of smallholder farmers, pastoralist or fisherfolks in these countries who produce and trade plant staples, fruits, vegetables, wild and domesticated livestock (McCullough et al. 2010; Lowder et al. 2016). These producers commonly sell onto local or regional markets through a series of (often but not always informal) “middle men” (aggregators, wholesalers and brokers) (Porter et al. 2007; Veldhuizen et al. 2020). Further down along the supply chain, the retailing segment is also dominated by informality, both in the structures (open markets, street vending, and corner stores) and in the transactional process (informal contracts, and agreements) (Cadilhon et al. 2006; Roever and Skinner 2016; Smit 2016). Local food systems feed the majority of the rural and urban population in LMICs, a large number of which are living in informal settlements under or close to the poverty line and spending more than 50% of their total income on food (Minot et al. 2013). As such, those local food systems are often the only source of affordable, nutritious food for both rural and urban poor communities.

High exposure and vulnerability to shocks affects most of those different groups of actors, essentially due to the small or micro-scale of their operations, the informality nature of the structure and contracting process, the lack of access to insurance system and to sufficient cash flow, the economic marginalization, and, in some cases, discrimination and harassment that affect these actors (e.g. street vendors in Vietnam, Kawarazuka et al. 2018), the predominance of women in systems still controlled by men (Kusakabe 2016), and the absence of labour protection and laws, facilitating exploitation, forced and child labour in production and processing sectors (Marschke and Vandergeest 2016).

## Impact of the COVID-19 on local food systems’ actors, an overview

There is still very little formal analysis of the impact of COVID-19 on local food systems and their actors. Although several special issues are expected to be available in the coming months, most of the information available at the time of writing (May–June 2020) derives essentially from web-based material, grey literature, news and social media accounts and first hand observations. In a period where the concept of fake news is a reality and the situation is evolving on a daily basis, providing an accurate and/or comprehensive description of the crisis, its severity and dynamics is therefore delicate. Table 1 is an attempt to synthesize the different types of adverse effects as they have been reported by various sources on the different actors operating in local food systems, and the subsequent (assumed or real) impacts on the food security dimensions (availability, access, quality and stability) of those actors. Due to space limitation, the content of Table 1 is not repeated in the text. Instead some ‘high-level’ conclusions are highlighted.

**Table 1:** Adverse impacts of the COVID on local food systems’ actors and expected direct effects on their food security

Actors	Types of adverse impacts reported	Expected *direct* effect on actors’ food security	Subsequent *indirect* effect on other actors’ food security
Producers (e.g. family-based farming/dairy enterprises)	▪ Disruption in input supply chain (e.g. fertilizer) and/or subsequent increase in input prices ▪ Reduction in demand of certain products (excess supply) leading to drop in farm-gate product prices ▪ Reduction in labour/workers availability (due to mobility restriction, increase in public transport costs, or fear of exposure to virus)	▪ Drop in profitability affecting producers’ income, purchasing power and access to traded food	▪ Reduced food availability for retailers, vendors and eventually consumers; disruption or reduced stability of food availability
Transporters (small to medium-sized enterprises)	▪ Transport affected by local or national mobility restrictions and lockdowns (e.g. time when they are allowed to travel on road) ▪ Increased risk of exposure to the virus	▪ Drop in profitability affecting transporters’ income, purchasing power and access to traded food	▪ Reduced food availability and food access for retailers, vendors and consumers; disruption or reduced stability of availability and access
Processors (formal or informal micro, small or medium-sized enterprises)	▪ Reduction in demand of certain items (excess supply) leading to decline in business profitability ▪ Shift in food suppliers (with potential drop in quality / stability of food traded)	▪ Drop in profitability affecting processors’ income, purchasing power and access to traded food	▪ Increase in risk of food safety issues for consumers
Retailers (formal or informal micro to small enterprises)	▪ Substantial increase in input costs leading to decline in business profitability ▪ in food suppliers (with potential drop in quality / stability of food traded)	▪ Drop in business, reduced income affecting retailers’ purchasing power and access to traded food	▪ Disruption of food supply chain ▪ Increase in risk of food safety issues for consumers
Vendors (e.g. street vendors, workers in small formal or informal food outlets and shops)	▪ Temporary loss of job or income due to lockdown and mobility restriction or (partial or total) closure of open air market ▪ Policy violence against informal street vendors ▪ If still operating, increased risk of exposure to the virus ▪ Decline in demand (due to drop in consumers’ purchasing power (see below) leading to fall in business profitability	▪ Drop in business, reduced income affecting vendors’ purchasing power and access to traded food	▪ Disruption of food supply chain affecting food availability ▪ Shift of consumers to more expensive food outlet (e.g. supermarkets)
Consumers including member of the other groups of actors of the food system (who are also consumers), and non-food system actors.	▪ Temporary loss of job and income due to lockdown and mobility restrictions ▪ Increased in costs related to food purchase (cost of transportation, cost of delivery, price of food) ▪ Disruption in access to food outlets of choice (lockdown affecting consumers mobility and access to food supply outlets) ▪ Disruption in food supply chain ▪ Loss of access to cheap, close-by, convenient food supply outlets (e.g. open air markets forced to close)	▪ Reduced income/wages affecting consumers’ purchasing power and subsequently access to food, with possible degradation in food quality (e.g. shift to cheaper, less nutritious food), or reduction in food purchase ▪ Reduction in stability of access to food ▪ Increased risk of exposure to unsafe food ▪ Forced shift to more expensive food outlets (e.g. supermarkets) leading to further fall in purchasing power	▪ Reduced demand for certain food items leading to reduction in income for vendors, retailers, and eventually producers

Although the focus of this review is on the disruptions induced by the 2020 COVID-19 pandemic on food systems and the implications on food security – paradoxically, we also need to keep in mind that the agro-food industry is actually one of the very few sectors that have been actively protected by governments and local authorities, compared for instance to other sectors such as air/sea travel, automotive industry, construction sector, or tourism/hostelry. Farmers, food suppliers, and other workers involved in the agro-food sector (transporters, processing factory or food outlet workers) are amongst those who are generally exempt from lockdown and working/mobility restrictions (with however some social distancing directives).

Despite this relative protection, Table 1 highlights a certain number of adverse effects on food system actors. Not all those effects are observed simultaneously, however, and not all are observed in the same place / food system, or affect every actors in one group the same way or with the same severity. Some generic patterns emerge however from this descriptive overview.

One important first conclusion is that - with the notable exception of those who lost members of their family to the virus- the major direct effect of COVID-19 on food system actors and their food security is through its impact on the income and associated purchasing power of all those actors induced by mobility restriction and lockdown, and the subsequent negative effect this has on their access to traded food. The possible implications of this decline in purchasing power are well-established in the literature: fall back into poverty, with negative mid- to long-term effects on (child) nutrition, deterioration of wellbeing and physical and mental health, etc. See Table 1 and Devereux et al. (2020 this issue) for a more in-depth discussion on this point.

The other dimensions of food security (availability, quality, stability) are also present in Table 1; for instance in some particular cases the availability may become an important issue when e.g. local urban open-air or wet informal markets are forced to close due to local restrictions and the (poor) consumers have then to depend on more distant (and possibly more expensive) formal food outlets (e.g. supermarkets). In some other cases, stability may be an important problem when for instance the food supply chains of particular items are disrupted. But in the great majority of cases reported as of today, the main impact seems to be related to the loss of purchasing power of those actors as consumers, not because the prices of food items has increased –although it has in some cases- but rather because their own income/wage has decreased or their ability to access cheap food has been disrupted.

Another aspect –to which we shall come back later in this paper- is the ‘ripple effect’ which is observed across food systems, that is, the fact that when one group of actors is affected, the effect rarely remains confined within this group and usually spills over either ‘downstream’ to the next actors along the supply chain, or sometimes ‘upstream’, for instance when the restrictive mobility measure (lockdown) affecting consumers reduces the demand for particular food items and affects back the other actors (vendors, retailers and eventually producers). The occurrence of this ripple effect is captured in the last column on the right hand-side of Table 1.

## Lessons from recent resilience research and relevance for the COVID-19 crisis

In the last 5 to 7 years, important progress have been made in the academic literature in relation to the concept of resilience and its measurement in the specific context of food security and humanitarian interventions (see e.g. Frankenberger and Nelson 2013; Winderl 2014; Béné et al. 2014; Serfilippi and Raminath 2018; Ansah et al. 2019 for some earlier reviews). Largely guided by the work of the WFP/FAO “Resilience Measurement Technical Working Group” (Constas et al. 2013 and subsequent technical papers^2^) this literature differs substantially from the wider literature on socio-ecological or psychosocial resilience by its specific focus on the effects of disasters and other adverse events on people’s food security (von Grebmer et al. 2013; d’Errico et al. 2018; Smith and Frankenberger 2018; Mercy Corps 2020). We propose to rely on some of the main conceptual progress that have been made in this nascent literature to identify specific ‘principles’ and lessons, which, we argue, are useful to improve our understanding and eventually our capacity to design interventions that can strengthen the resilience of local food systems in the context of COVID-19 and beyond.

### Better understanding food system actors’ responses in the face of COVID-19

Resilience is notoriously difficult to quantify – essentially because it is a latent variable, that is, a variable that cannot be directly observed and measured (in contrast to, for instance, income poverty, malnutrition, or land ownership) (Constas et al. 2013; d’Errico et al. 2016). In those conditions, academics and practitioners interested in monitoring or measuring resilience are left with two alternatives: either to rely on some form of proxies that are thought to reflect indirectly the level of resilience (e.g. Smith et al. 2015; FAO 2016), or use information derived from self-assessed resilience measure, using psychometric techniques (Nguyen and James 2013; Béné et al. 2016a).

**Fig. 1:**
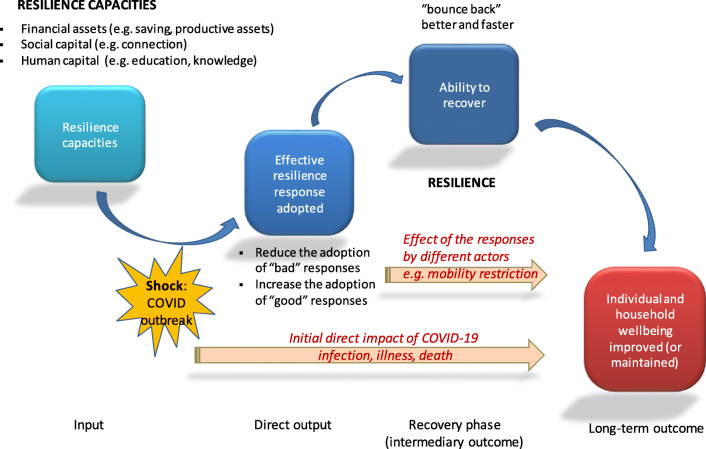
Resilience causal pathway and the impact of COVID (modified from Béné et al. 2015)

To understand better this measurement issue, we propose to examine the steps that form the generic causal pathway of resilience and see how the outbreak of COVID-19 affects those steps. Figure 1 illustrate this generic pathway conceptualised at the level of individual household. This household can be a farmer, or any actor within the food system (e.g. a family involved in processing, in retailing, in selling, etc.) or even a consumer.

Resilience is now often understood as resulting from a set of *capacities* or *abilities* (see e.g. Béné et al. 2012a; Constas et al. 2014). These capacities, which are represented on the left-hand side of Fig. 1, depend essentially on a combination of assets and capitals (social, human, financial) that households can draw on in anticipation, or in response to a sudden shock or a recurrent stressor. Although there does not seem to be any ‘unique’ or ‘perfect’ combination, the current evidence suggests that for farmers, financial/assets and to a lower extent social capitals are key in this resilience process (e.g. Fafchamps and Lund 2003; Carter and Barrett 2006; Aldrich 2010; Woodson et al. 2016). It would be important to explore if this general pattern is also observed for the other actors of the food systems or if different types of capacities are more specifically critical for other groups of actors.

Another important resilience principle that emerged from the recent literature is that, in the face of shocks, households will use these assets/capitals to develop adequate strategies/responses. By *adequate*, we mean strategies that reduce the risk of inducing harmful mid- or long-term consequences, and instead increase the chance to lead to ‘positive’ outcomes. In that sense (like in the psychosocial literature), resilience in the context of food security has been given some normative dimension; it is about the alternative (‘good’ or ‘bad’) responses that actors can, or cannot, engage in when faced with a specific adverse event. For smallholders at the upstream end of the food supply (farmers, small-scale fishers, agro-pastoralists) harmful or unsafe responses generally correspond to what has been long labelled in the literature as negative coping strategies (Corbett 1988; Devereux 1993; Kazianga and Udry 2004; Hoddinott 2006) such as selling productive assets, borrowing money, or reducing health, education or food expenses / consumption. In the case of COVID-19 we saw in Table 1 that when affected by a decline in income induced by the introduction of mobility restriction or the temporary loss of job due to lockdown regulation, households may have no choice but to reduce food expenses or to shift to cheaper but lower-quality food.

Those detrimental strategies can turn out to be, however, harmful not only for the members of the household who adopt them, but for other actors along the chain, or for the environment. Generic examples include the spreading of pesticide by traders or sellers to increase the market “longevity” of their products (leading to food safety issues) (Spanoghe 2017); or engaging in overfishing/deforestation/overgrazing activities to make up for a drop in revenues (Ferse et al. 2012; Smith et al. 2016). For other actors such as retailers or street vendors, examples of detrimental strategies would include shifting to unhealthy or unsafe (but cheaper) supplies to maintain their benefit margin when faced with a drop in consumers’ demand/presence or when trying to cope with an interruption in food supply. Those last examples which are already observed in some countries in the case of the COVID-19 crisis, can also occur for other types of shocks or stressors such as the interruption in supply following a disaster (e.g. a local flood) or the impact of recurrent armed attacks on local economy (Reddy et al. 2016). At the consumer level, shocks or stressors that increase consumer’s sense of uncertainty may trigger negative behaviour such as hoarding and panic buying –as it was observed in the first few weeks following the outbreak of COVID-19 and the subsequent disruption it incurred in local food supply chains (Lewis 2020; Norberg and Rucker 2020).

At the other end of the spectrum, more ‘positive’ responses would be those that help actors anticipate, better adapt or mitigate the impact of the shocks. For producers/farmers affected by the COVID-19 crisis, this would include (for instance) the capacity to rapidly shift to other input suppliers when their usual supplier announced an interruption in their own imported supply; or the ability to find substitute workers to replace the contracted ones who have been unable to come on site because of strict lockdown regulations where they live, or increased in public transport costs, or even fear to be exposed to the virus.

For other types of shocks or stressors such as those related to weather-extreme events (e.g. droughts, floods, typhoons, etc.) positive strategies for farmers include those that have long been identified and documented in the climate change adaptive literature (Arslan et al. 2015; Himanen et al. 2016; Abdul-Razak and Kruse 2017). For other actors, the understanding, knowledge and evidence about what constitutes a ‘positive’ answer is much thinner. “The resilience of food systems is not consistently assessed and hardly synthesized for low- and middle-income countries” (Meyer 2020, p.1). Very little is known, therefore, about what strategies/interventions would strengthen the ability of processors, or traders, or street vendors to react (or anticipate) positively to shocks or stressors, especially if those actors are operating in LMICs (Kawarazuka et al. 2018; Meyer 2020). The empirical literature on market actors’ resilience is factually non-existent and the lack of data that characterizes this “missing middle” is very disabling, especially in LMICs (Veldhuizen et al. 2020).

One reasonable approach to address this knowledge gap would be to extrapolate what works for farmers; we would still have to test whether the principles that underpin those farmers’ strategies also work for the other actors of the systems. For instance while it is often assumed that being connected to the market is an important prerequisite for farmers to strengthen their resilience (e.g. Meuwissen et al. 2019; Kangogo et al. 2020), we also know that too much connectivity is likely to expose people to “concatenated shocks” (Biggs et al. 2011). The outbreak of COVID-19 is a vivid illustration that this observation applies not just to farmers but to the entire food system. Another important part of the literature discusses the role of risk perception and other psychosocial factors such as aspiration, self-efficacy, personal experiences with extreme weather events and how those factors affect farmers’ adaptive capacity (e.g. Grothmann and Patt 2005; Boissiere et al. 2013; Van der Linden 2014; Eitzinger et al. 2018). Similar effort have been conducted recently in relation to households’ resilience in the context of food security crisis (e.g. Jahan et al. 2015; Béné et al. 2019a). Those authors found that a higher sense of self-efficacy for instance reduces the chance of households to engage in detrimental copying strategies. It would be useful to explore whether those findings also apply to the other actors of the food system. The example of hoarding and panic-buying mentioned earlier would typically be the type of behaviour which could be more systematically analysed with those approaches in order to determine the role that subjective factors such as risk perception or individual or collectively constructed sense of locus of control^3^ (Lefcourt 1991) do play a role in the decision making process of these actors when faced with shocks.

A complementary approach would be to explore some of the principles that have been identified in the literature on value chain and agribusiness supply chain. Although a substantial part of that literature focuses on global/international value chain operations and on formal/modern agribusiness supply chains (e.g. Gereffi et al. 2005; Behzadi et al. 2018; Kano 2018), some principles identified there may be relevant for more local, informal food system actors. For instance, the importance of inclusiveness in value chain is almost universally recognized as an important element to improve the economic viability or even the long-term sustainability of business (Helmsing and Vellema 2010; Kilelu et al. 2017). Most of those discussions have been conducted, however, outside the resilience realm^4^ and no specific attention was given to the question of shocks –even though ‘disruption’ is a relatively well-established concept in relation to value chain leanness (e.g. Behzadi et al. 2017). One would have, therefore, to test whether this principle of inclusiveness also increases the likelihood of local food system actors to engage in more positive responses while reducing their propensity to adopt negative strategies -and thus, contributes to strengthen the resilience of local food systems.

Table 2 presents some of the principles that have been discussed in either the farmer’s climate change adaptive literature or the value chain literature, and which would need to be explored more systematically in the case of local food systems’ resilience. The most frequent ones include: diversification; substitution; entrepreneurship; cooperation; competition; connectivity; index-based insurance; inclusiveness; cash transfer, and subjective resilience. Those are listed in the left-hand side of Table 2. The column of the right-hand side indicates how some of those principles could contribute to build the resilience of local food system actors in the light of the types of COVID-19 disruptions as described in Table 1. Those mitigating effects are hypothetical however and would need to be tested empirically.

**Table 2:** Principles of risk management strategy discussed in the farming system and/or the supply chain literature, and are of potential relevance for local food system resilience in the context of the COVID-19 crisis

Principle	Definition	References	Potential positive effect in the case of COVID-19 (to be empirically tested)
Diversification	The ability of actors of the food system to changes the set of products (crops, raw or processed products, etc.) that they offer to the market, or the actors from whom they obtain their inputs/food supplies.	Ramasesh et al. 1991; Backus et al. 1997; Arslan et al. 2015; Tukamuhabwa et al. 2015; Cunningham and Jenal 2016; Barot et al. 2017; Reed et al. 2017	Diversification could reduce the level of disruption in supply chains faced by producers and other actors along the food supply chain (processors, retailers, sellers, etc.), thus mitigating the negative effects that these disruptions have on their operations and incomes.
Substitution	The degree to which the different food system actors can have access to input products that are similar or comparable (in terms of price, quality, or characteristics e.g. nutrition value)	Ganesh et al. 2008; Goyal and Netessine 2011;	Substitution would reduce the disruption effects on supply of certain inputs in food processing, or on the availability of food items for consumers, thus mitigating the negative effects that those disruptions have on food system operations and consumers’ food and nutrition.
Entrepreneurship	Refers to actors’ behaviour when they proactively adapt, take calculated risks, and innovate in response to stimuli from both internal and external environments.	Iza et al. 2019; Kangogo et al. 2020	Entrepreneurship would improve actors’ ability to anticipate and respond to shocks or stressors. In the case of COVID-19, example would include those retailers or vendors who rapidly established safe food delivery services and in so doing reduced the risk of infection amongst some at-risk populations (e.g. elderly).
Cooperation	Cooperation is an outcome of social capital; it refers to situations in which food system actors (within and across socioeconomic groups: producers, traders, street vendors, etc.) seek out win-win outcomes from working together.	Rose 2014; Downing et al. 2018	Cooperation within or between groups of food system actors would reduce the negative effects of mobility restrictions imposed by local or national authorities. For instance better cooperation between farmers and workers could help reduce the drop in labour supply.
Competition	Competition is expected to stimulate actors of the food system to develop new products, services and technologies, which would give consumers greater selection and better products.	Gorodnichenko and Roland 2012; Downing et al. 2018	Competition between actors within the same groups (e.g. retailers) would stimulate the supply of better quality or more affordable food products, thus mitigating the negative effects of food supply chain disruptions or loss of income on consumers’ food security.
Connectivity/ farmer–buyer relationships	Connectivity refers to the intensity and nature of the relationships (vertical, horizontal, positive, negative) between different actors within and across socio-economic groups (farmers, traders, processors, etc.)	Frank and Penrose-Buckley 2012; Goerner et al. 2009; Downing et al. 2018; Kangogo et al. 2020;	Like diversification or substitution, connectivity would reduce the disruptions faced by producers and other actors (processors, retailers, sellers, etc.) along the food supply chain, thus mitigating the negative effects that these disruptions have on their operations and incomes.
(Index-based) insurance	Index-based insurance refers to insurance contracts used (so far) essentially in farming systems where payouts are based on an index (e.g., rainfall, yield or vegetation levels) that is correlated with agricultural losses.	Bertram-Huemmer and Kraehnert 2015; Hill et al. 2017	Index-based insurance could be used to protect food system actors from specific shocks affecting their businesses, thus reducing their propensity to engage in negative responses. In the case of COVID-19 access to these index-based insurance could have reduced the risk of, e.g., vendors having to break authorities’ order and continue operating in crowded informal markets in order to secure some minimum income.
Inclusiveness (economic or gender inclusion)	Inclusive value chains usually place emphasis on identifying ways in which low-income actors (male or female) can be “better” incorporated into existing or new value chains or can extract greater value from the chain.	Goerner et al. 2009; Helmsing and Vellema 2010; Kilelu et al. 2017; Downing et al. 2018	Making local food systems more inclusive would mean offering food supply informal and micro-enterprises more opportunities to build their resilience capacities (better networking, better access to infrastructures better access to information, better protection/insurance, etc.). In the case of COVID-19, those various capacities would have helped those small actors to be better prepared (sometimes simply by having more savings) to face the COVID-19 disruptions.
Cash transfer	Cash transfers refers to social protection interventions whereby a direct payment of money (cash or electronic transfer) is made to an eligible person (i.e. one that satisfies a certain combination of criteria).	Gilligan et al. 2009; HLPE 2012; Béné et al. 2012a, 2012b; Davies et al. 2013; Soares et al. 2016; Béné et al. 2018; Asfaw and Davis 2018	Distribution of cash during the weeks/months during which households are forced to stop their economic activities due to lockdown is one of the most effective way to reduce the negative effect of COVID-19 crisis on the millions of actors (consumers, farmers, vendors, workers, etc.) who have lost their jobs temporarily or are facing a reduction in their incomes.
Psychosocial factors and subjective resilience	Psychosocial factors such as risk-perception, self-efficacy, aspiration, or perseverance are recognized to contribute to people’s construct of subjective resilience and influence their choice of responses in the face of adverse events	Bernard and Seyoum Taffesse 2014; Jahan and Wahab 2015; Béné et al. 2019a	Boosting the self-confidence, self-efficacy and aspiration of people has been shown to have positive effect on their ability to engaging in constructive responses when faced with adversity. Implementing interventions that improve the perception that actors have about themselves and their capacities to deal with hardship (self-efficacy) is something that government and development agencies should envisage to strengthen the resilience of local food systems.

### Food system resilience: COVID-19 crisis and beyond

As a conceptual framework, Fig. 1 highlights important additional lessons that can be useful to understand better how to build the resilience of food system actors in response to the COVID-19 crisis. Some of those lessons go beyond the specific case of the pandemic, however. In this section, we propose to discuss four of those lessons, which, we argue, are relevant not only for improving our understanding of local food system resilience in the face of COVID-19 but also for other types of adverse events classically observed having substantial impacts on food systems and their actors, including extreme weather related events (drought, flood, natural disasters), economic or political crises (trade ban, economic collapses), etc.

#### Lesson 1: Distinguishing resilience from resilience capacity

Altering actors of food systems’ propensity to engage into specific strategies (helping them in particular to adopt ‘positive’ responses and reducing their propensity to engage in detrimental ‘negative’ strategies), is expected to help them strengthen their *actual* resilience, that is their capacity to bounce back better and/or faster than they would otherwise. This critical component (the resilience per se) and the associated step (the recovery phase) is shown in the resilience causal model on the right-hand side of Fig. 1. In the long-run, this ability to recover more efficiently is what helps people restore, protect, maintain (or, in some case, improve) their levels of wellbeing in the face of shocks.^5^

In that causal model, resilience capacities are the different elements, tangible or less tangible, that actors of the food system have at their disposal, which they have accumulated, built, developed (income, knowledge, social capital, etc.) and that they may or may not use in response to a crisis/shock. In contrast, resilience is their *actual* ability to recover (to bounce back) from that crisis/shock. Although related, these are two distinct concepts, corresponding to different steps along the resilience pathway (Béné et al. 2015). Put simply, resilience capacities are *input* to the resilience process, while resilience per se is the (intermediary) *outcome*, contributing to the longer-term final outcome (which itself is measured in terms of wellbeing). Yet, too often, people conflate resilience and resilience capacity. Part of the reasons for this confusion is the difficulty to measure resilience per se. Because it is easier to measure elements such as level of savings, assets, or access to health centres or infrastructures than it is to measure the capacity to recover from shocks itself, researchers or practitioners are often tempted to claim that they are measuring resilience, whereas what they measure are in fact indicators of resilience capacity. For instance, using the five sustainable livelihood capitals (financial, natural, physical, social, and human) as a proxy for resilience would be contributing to this conceptual confusion (see e.g. Thulstrup 2015; Quandt 2018).^6^

For those amongst academics, practitioners and policy makers interested in better understanding the dynamic of food system resilience and in establishing how food system resilience eventually affects the wellbeing or food/nutrition security of the different actors within the system, it is important to ensure that the two concepts (resilience capacities and resilience per se) remain distinct and are measured separately. On one hand, estimating resilience capacities would involve using quantitative or semi-quantitative indicators that are usually available from conventional individual socio-economic surveys or focus group discussions. These include household levels of assets or savings; education and experience; access to information, public services and infrastructures; social network and other social capital indicators, etc. (Downing et al. 2018). The list of those resilience capacity indicators is not limited and the choice should be guided by the underlying hypotheses driving the research. For instance, one may hypothesize that in the context of informal food systems, access to better-tailored information on food safety issues is critical but not sufficient to reduce the propensity of traders to use pesticide on their products and that accompanying interventions around psychosocial factors (e.g. women self-efficacy) may be necessary to build efficiently the *resilience capacity* of those informal actors. In that case, indicators capturing those two different types of capacities (access to information and women self-efficacy) would need to be included in the project’s monitoring and evaluation system.

On the other hand, establishing whether this increased resilience capacity translate in effective resilience when those actors are impacted by a specific shock would require a different type of data/approach. Measuring resilience per se is challenging and no consensus has been reached at the present time on how to measure it. Several frameworks and approaches have been proposed in the context of humanitarian and food security interventions (see e.g. Winderl 2014; Schipper and Langston 2015; or Serfilippi and Raminath 2018 for some recent reviews) but none of those frameworks has been developed with food system resilience as the object of the study. Instead, they all use household or community as their unit of analysis (see e.g. Cutter et al. 2008; Cohen et al. 2013; Smith et al. 2015; Béné et al. 2016b; FAO 2016; D’Errico et al. 2018). In several cases, the difficulty of measuring resilience per se is avoided by considering the next stage along the pathway, and measuring the outcome of resilience, using food security or nutrition indicators (e.g. d'Errico and Pietrelli 2017; Smith and Frankenberger 2018). The only few cases where resilience per se has been measured directly is through self-assessed recovery index estimated through series of recall questions and psychometric techniques (e.g. Nguyen and James 2013; Béné et al. 2017; Béné et al. 2020) in a similar way it is done in psychological resilience literature (see Windle 2011 for a review). It would be important to pursue those different approaches in the context of food system actors.

#### Lesson 2: Measuring not just resilience but its long-term outcomes as well

In the humanitarian and food security literature, household long-term wellbeing is often proxied by food security and/or nutritional status indicators such as the Household Food Insecurity Access Scale (HFIAS) (Coates et al. 2007), the Household Dietary Diversity Scores (HDDS) (Swindale and Bilinsky, 2006) or the z-score of individual member of the household (WHO 2006).^7^ Technically, the HFIAS is essentially looking at the access dimension of food security while the HDDS is focusing on the utilisation dimension.

Other indicators are sometimes used in the literature, such as level of income or assets (e.g. Carter et al. 2007; Cissé and Barrett 2016), but those are more problematic as income and assets are also considered to be part of resilience capacity – which would put them on both sides of the equation when one tries to correlate levels of resilience capacities with long term outcomes.

For food system resilience, the levels of the consumers’ food and nutritional security are obviously two very important outcomes and it seems logical that indexes of food security and nutrition such as HFIAS or HDDS remain amongst the indicators used for assessing food system resilience. Observing for instance a rapid deterioration in the HFIAS of urban dwellers in a region that has been affected by recurrent armed attacks or by a local flood would indicate a system that has a poor resilience to those specific shocks. However, the disruption of food systems’ operations may result in other forms of detrimental impacts. The direct impact of a landslide or a drought may not just lead to the interruption of supply –affecting the availability dimension of food security. It is likely to affect also the access/affordability of food items (Islam and Al Mamun 2020) leading to subsequent rise in local food price or deterioration in the quality of the items traded, with potential issues of food losses (food products damaged by flood for instance, Reddy et al. 2016); food safety (mycotoxins/aflatoxins contamination due to too long storage in humid/unsanitary conditions, Liu et al. 2016) or even nutrient leakages (heat sensitive micronutrients deteriorating quickly when exposed to high temperatures).

In that context, using the HFIAS or even the HDDS index as it is often done for household resilience would not permit to capture the entire range of potential disruptions that can affect a food system and to assess the different dimensions attached to its (lack of) resilience. We would also need to ensure that indicators specific to those other dimensions are also included in the analysis. Additionally, because food systems involve different groups of actors (as opposed to just producers or consumers) those different potential impacts (fluctuation in supply, loss in food quality, risk of contamination, nutrient leakages) would have to be assessed for all the different actors along the supply chain. Some of the links/actors may be more (less) resilient than others, and some of the impacts may be actor-specific.

Finally, analysing the stability over time of those different indexes for those different groups of actors would also be necessary, as it may be that a specific shock alters not the average value of an indicator over time –say, the quantity of food supplied to a market, or its average price-, but the volatility/instability of that indicator.

Those different points are summarizes in Table 3. The first column (on the left-hand side) lists some of the key indicators that were discussed above and that should be included in a food system resilience analysis when assessing specific long-term outcomes. The second (middle) column indicates the food security dimensions which these indicators relate to; it shows that the four dimensions of food security (availability, accessibility, quality and stability) could in theory be assessed using appropriate indicators. The third column (on the right-hand side) indicates what actor groups those indicators are expected to provide information about. It shows that while some indicators are generic and can be used in relation to any group of actors within the food system (producers, processors, sellers, etc.), other are more specific to particular groups. An important conclusion that emerges from Table 3 is that no indicator is capable to cover comprehensively and simultaneously the four food security dimensions and all the different groups of actors.

**Table 3:** Examples of indicators susceptible to be used to assess long-term outcomes of food system resilience

Indicators of long-term outcomes	Food security dimensions	Actors
Household Food Insecurity Access Scale ^a^	Food Access	Any consumers within the food system
Household Dietary Diversity Scores ^b^	Utilisation - Food Quality	Any consumers within the food system
z-score ^c^	Utilisation - Food Quality	Any consumers within the food system
Post-harvest contamination with mycotoxins ^d^	Utilisation – food safety	Producers – processors - sellers
Post-harvest losses ^e^	Availability	Producers - Processors
Nutrient leakages ^f^	Utilisation - Nutrition	Producers – Processors - retailers
Presence of pesticide in food products ^g^	Utilisation – food safety	Producers – processors - sellers
Price volatility index ^h^	Food access / Stability	Any actors within the food system
Food waste ^i^	Availability	Consumers

^**a**^: Coates et al. 2017; ^b^: Swindale and Bilinsky, 2006; ^c^: WHO 2006; ^d^: Magan and Aldred 2007; ^e^: FAO 1994; ^f^: FAO 2011; ^g^: WHO 2001; ^h^: Díaz-Bonilla 2016; ^i^: EPA 2014

#### Lesson 3: Long-term resilience outcomes don’t result solely from shock’s direct impacts

The third major lesson illustrated in Fig. 1 which is relevant to improve further our understanding of food system resilience is that the final outcome of the resilience causal pathway (be it measured in terms of consumers’ food or nutritional security, or loss of nutrient) does not result merely from the direct impact of the initial shock (e.g. destruction of crops, losses of livestock, or disruption in supply due to import bans), but from the combination of the direct impact(s) of that shock with the responses that actors (individually or as groups) put in place to mitigate or counteract that shock. This point is illustrated on Fig. 1 by the presence of the two arrows “Initial impact of the shock” and “Effect of the responses”. While this conclusion was already important in the analysis of farming households, it becomes even more important for the analysis of food system resilience. The painful experience of the impact of COVID-19 on food systems illustrates perfectly this point: the current threat to the food security of millions of people in the world is not the direct effect of the virus itself, but the results of the disruptions in food supply and in income revenues induced by the restriction of movement imposed by national/local governments (Table 1 and Devereux et al. –2020 this issue). Those restrictions were themselves the attempts by those authorities to respond to the initial health impact of the pandemic. The negative effects of these restrictions were in some cases further exacerbated by other actors’ responses, such as panic buying and hoarding (Lewis 2020). Here again, the eventual impact on other people’s food security was not the result of the initial shock (the virus) but the consequent of the ‘maladapted’ responses adopted under panic by some of the system’s actors (Norberg and Rucker 2020).

#### Lesson 4. Recognizing the importance of ‘ripple effects’

**Fig. 2:**
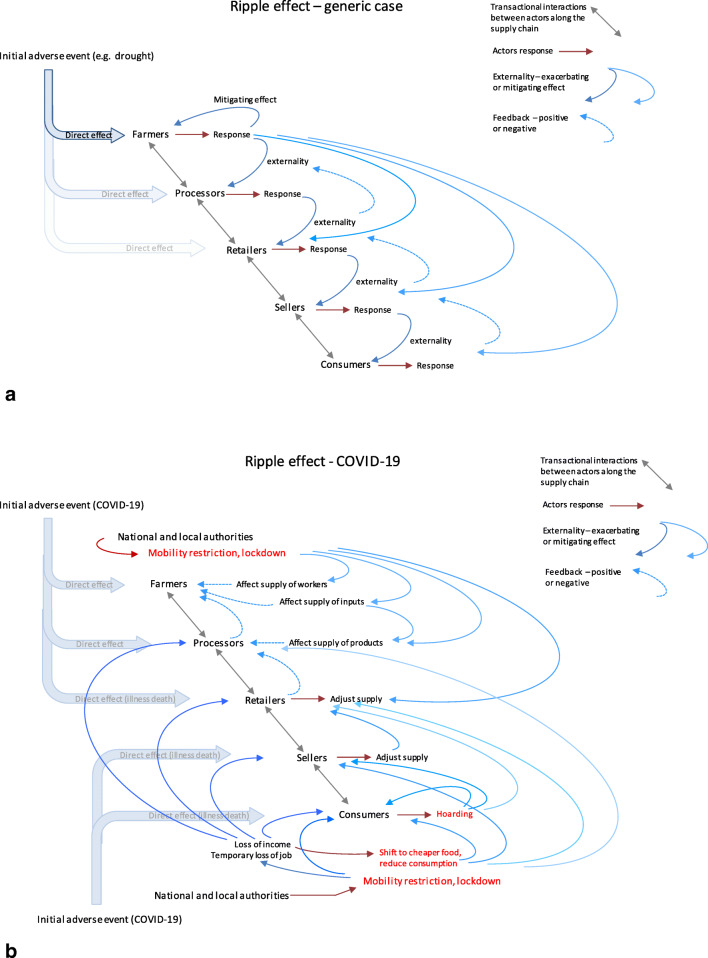
**a** Ripple effect of responses throughout the supply chain (generic case). An initial shock (here a local drought) which direct effect may be restricted to the first groups (farmers and processors) may trigger responses and feedback effects all the way down to the consumers affecting everyone along the supply chain. **b** Ripple effect of responses throughout the supply chain in the case of the COVOD-19. Here the major sources of externality are the mobility restriction and lockdown imposed by the authorities which trigger major ripple effects throughout the food system, downward from the producers and upward from the consumers

A corollary to Lesson 3 is that, in order to conduct a comprehensive food system resilience analysis, it is not enough to document the nature, severity and duration of the different shocks/stressors that can potentially affect a food system and the subsequent levels of exposure, sensitivity and vulnerability of the different actors to those various shocks. It is as important (or perhaps even more important) to document and analyze the types of responses that the different groups of actors put in place as an attempt to mitigate the effects of those various shocks, and to assess carefully the potential positive and negative “externalities” that those responses generate on people’s own wellbeing but also on the other actors of the system. This finding, which had already been emphasized in the ‘simpler’ case of farming households or communities for which it was stressed that the “resilience of some may be built at the detriment of others” (Béné et al. 2016a, p.130),^8^ is even more true for food systems where the interconnections and dependency within and between groups of actors is likely to create a potentially very powerful ‘ripple effect’ throughout the food system (Fig. 2a).

The existence of this ripple effect is possibly one of the major differences between assessing the resilience of households or community, and assessing resilience of food systems. The very nature of food systems, made of interconnected actors and feedbacks (Ericksen 2008; HLPE 2017; Béné et al. 2019b), means that once an initial shock impacts one part/group of actors in the system, the responses it triggers from that group is likely to ripple through the interconnected parts/groups, often in an unpredictable way. The final outcomes of those exacerbating or mitigating effects are made even less predictable by the existence of positive or negative feedback loops (when the strategies put in place by some actors to respond to the initial responses trigger subsequent responses by other actors), leading to even more unpredictable and unintended consequences. In that context, what policy makers aiming at strengthening the resilience of local food systems would seek is to foster synergies and ‘virtuous spirals’ of positive responses and negative feedbacks^9^ to reduce the chance of harmful and catastrophic unintended consequences. Figure 2b represents the ripple effect of COVID-19 crisis, illustrating (by contrast with Fig. 2a) the specificity of those effects and thus reiterating the importance of documenting them precisely if one wants to be in a position to build food system resilience.

## Concluding remarks

In this paper we were interested in exploring the concept of food system resilience in the light of the disruptions brought to those systems by the COVID-19 pandemic. For this, we used the most recent advances made in the literature on household/community resilience in the context of food security and humanitarian crises and identified how these lessons can be used to improve our understanding of the impact of the COVID-19 crisis on local food systems and its implications on people’s food security. The discussion was broadened, however, to consider other shocks and stressors beyond COVID-19: extreme weather related events –drought, flood, natural disasters– but also social, economic or political disturbs (price peak, trade ban, local insecurity, etc.).

One of the first conclusions that emerged from this analysis is the recognition that the current threat to the food security of the millions of people affected by the COVID-19 crisis is not the result of the virus itself (infection, illness, or death), but the consequence of the loss of income and purchasing power induced by the lockdown and shutting down of enterprises imposed by national/local governments. Translated in the four dimensions of food security conventionally referred to in the literature (availability, access, quality and stability), this means that notwithstanding the few cases of reported disturbances on food availability and stability (induced by disruptions in transports or resulting from temporary hoarding behaviour), most of the impacts of the COVID-19 have been until now (June 2020) mainly around the access dimension of people’s food security: “when individuals and households have [not] adequate resources to obtain appropriate food” (FAO 2008).

From a food system resilience perspective, a couple of key-points were highlighted. The first one builds on the observation made just above: it stresses that to be able to capture issues around food system resilience it is imperative not to focus just on the initial impact of the shock (destruction of crops, export ban, price peak, or in the present case, impact of the COVID-19 virus on people’s health) but to also incorporate in the analysis the different responses adopted by the different actors –including policy makers. In other words, the ultimate ability to bounce back and recover from a shock does not depend solely on the intensity/severity of the initial shock, but on the impact of that shock’s *combined* with the responses that actors (individually, or as communities or society) put in place to mitigate or counteract the initial effect of that shock –sometimes with unintended consequences.

Second, as it is for households or communities, building resilience in food systems is about building capacities. Assets, savings, access to insurance are probably keys in that respects. Likewise, diversification, connectivity, and substitution are likely to be important. But those are typically the type of principles that are discussed in the context of formal actors operating in formal context. For the elderly women selling fruits and vegetables on wooden racks in the streets of Kinshasa or for the men travelling at dawn more than 70 km by motorbike to supply eggs and chicken to their cousins on the wet markets of Hanoi, those are remote potentialities. For the large majority of the actors in LMIC’s local food systems, developing capacities that are more in line with the characteristics and informality of their environment will require more, well-designed, research. Very little is known about the resilience strategies of those actors. One can only assume that better access to information, stronger cooperation, more inclusion, and higher levels of aspiration and self-efficacy for those actors will go a long way, even if those are not as easily monitored changes as increased savings or number of markets built.

Finally, the economic, institutional and social relationships that exist between the different actors within food systems makes them intimately dependent on each other. Adopting a food system resilience framework helped better realise the complexity – and sometime very unstable nature – of the situation and the potential ripple effects that may pass through the entire food system once one component is affected. Analyzing –or anticipating– these ripple effects (being able to foresee their nature, dynamics, directionalities, etc.) should be an essential element of any food system resilience analysis in the future.

To conclude, the 2020 COVID-19 crisis revealed how unprepared the world was to respond appropriately to the pandemic. It showed in particular how decision-makers, from the international down the local levels, were poorly equipped to navigate the painful trade-off between health and economy, and how, as a consequence (and as it is often the case), the poor have been the ones who suffered the most from this. Soon (if not already) the “post-COVID” discourse will become the new reference, with its assortment of optimistic narratives whereby the world will be encouraged to turn the current crisis into an “opportunity to build back, better and stronger”. We are hoping those narratives will not be just another rhetoric and that some of the resilience principles discussed in this paper can contribute to the necessary changes.

## Footnotes

1 We will therefore not discuss macro-economic issues such as loss of GDP or disruptions in international trades.

2 https://www.fsinplatform.org/resilience-measurement

3 Locus of control, a correlate of self-efficacy, refers to the extent to which individuals believe they can control external events affecting them.

4 Note however that there is a relatively rich literature on supply chain resilience (see e.g. Martin and Peck 2004; Ponis and Koronis 2012) but this literature is generic, written for formal economy and does not focus on *food* supply chain.

5 Note that this ability is also influenced by many other factors, such as the level of external help/support received in the aftermath of a particular disaster/shock; but those are outside the control of the households. In the case of COVID-19, receiving cash transfer would be an example of those external interventions that can strengthen the ability of the actors to mitigate the effect of the COVID-19 crisis.

6 This confusion even affects systematic reviews (e.g. Patel et al. 2017) where the authors propose to review the concept of “community resilience” and identify what they refer to as “core elements of community resilience”. Those elements appear however to be resilience capacities: local knowledge, community networks and relationships, communication, health, governance and leadership, resources, economic investment, preparedness, and mental outlook.

7 One can also assess the mental wellbeing of households affected by shocks, using for instance Diagnostic and Statistical Manual of Mental Disorders DSM scale (see e.g. Fullerton et al. 2004) or even a more generic wellbeing index (e.g. the OECD Better Life framework, Boarini et al. 2014).

8 Pain and Levine (2012) for instance, in their analysis of livelihood trajectories in rural Afghanistan, reported a situation in which a landlord’s resilience is reinforced to the detriment of the resilience of his sharecroppers.

9 Although counter-intuitive and misleading, negative feedbacks are those mechanisms that avoid systems to ‘explode’ or ‘collapse’ by mitigating or reducing/buffering the effect of the initial responses. In contrary positive feedback loops are those which amplify further the initial signal/response and generally lead to catastrophic effects.
